# Redetermination of junitoite, CaZn_2_Si_2_O_7_·H_2_O

**DOI:** 10.1107/S1600536812037622

**Published:** 2012-09-08

**Authors:** Hexiong Yang, Neil G. Jenkins, Robert T. Downs

**Affiliations:** a Department of Geosciences, University of Arizona, 1040 E. 4th Street, Tucson, Arizona 85721-0077, USA; b Sabino High School, 5000 North Bowes Road, Tucson, Arizona 85749, USA

## Abstract

The crystal structure of the mineral junitoite, ideally CaZn_2_Si_2_O_7_·H_2_O (calcium dizinc disilicate monohydrate), was first determined by Hamilton & Finney [*Mineral. Mag*. (1985), **49**, 91–95] based on the space group *Ama*2, yielding a reliability factor *R* of 0.10, with isotropic displacement parameters for all non-H atoms. The present study reports a structure redetermination of junitoite using single-crystal X-ray diffraction data from a natural sample, demonstrating that the space group of this mineral is actually *Aea*2, which can be attained simply by shifting the origin. Topologically, the structure models in the space groups *Aea*2 and *Ama*2 are analogous, consisting of chains of corner-sharing ZnO_4_ tetra­hedra parallel to the *b* axis, cross-linked by Si_2_O_7_ tetra­hedral dimers (the site symmetry of the bridging O atom is ..2) along *a* and *c*, forming a three-dimensional framework. The Ca^2+^ cations (site symmetry ..2) are situated in cavities of the framework and are bonded to five O atoms and one H_2_O mol­ecule (site symmetry ..2) in a distorted octa­hedral coordination environment. However, some bond lengths, especially for the SiO_4_ tetra­hedron, are noticeably different between the two structure models. Hydrogen bonding in junitoite is found between the water mol­ecule and a framework O atom.

## Related literature

For junitoite, see: Williams (1976 ▶); Hamilton & Finney (1985 ▶). For junitoite-related minerals and compounds, see: Lin *et al.* (1999 ▶); Fleet & Liu (2001 ▶); Kolitsch *et al.* (2009 ▶); Yang *et al.* (2012 ▶). Parameters for bond-valence calculations were taken from Brese & O’Keeffe (1991 ▶).

## Experimental

### 

#### Crystal data

CaZn_2_Si_2_O_7_·H_2_O
*M* *_r_* = 357.02
Orthorhombic, 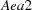

*a* = 12.530 (4) Å
*b* = 6.3056 (18) Å
*c* = 8.562 (3) Å
*V* = 676.5 (3) Å^3^
*Z* = 4
Mo *K*α radiation
μ = 8.21 mm^−1^
*T* = 293 K
0.06 × 0.06 × 0.05 mm

#### Data collection

Bruker APEXII CCD area-detector diffractometer
Absorption correction: multi-scan (*SADABS*; Sheldrick, 2005 ▶) *T*_min_ = 0.639, *T*_max_ = 0.684
2719 measured reflections
1257 independent reflections
1189 reflections with *I* > 2σ(*I*)
*R*_int_ = 0.021

#### Refinement

*R*[*F* ^2^ > 2σ(*F* ^2^)] = 0.017
*wR*(*F* ^2^) = 0.044
*S* = 1.07
1257 reflections
65 parameters
1 restraint
H atoms treated by a mixture of independent and constrained refinement
Δρ_max_ = 0.52 e Å^−3^
Δρ_min_ = −0.65 e Å^−3^
Absolute structure: Flack (1983 ▶), 580 Friedel pairs
Flack parameter: 0.023 (12)

### 

Data collection: *APEX2* (Bruker, 2004 ▶); cell refinement: *SAINT* (Bruker, 2004 ▶); data reduction: *SAINT*; program(s) used to solve structure: *SHELXS97* (Sheldrick, 2008 ▶); program(s) used to refine structure: *SHELXL97* (Sheldrick, 2008 ▶); molecular graphics: *XtalDraw* (Downs & Hall-Wallace, 2003 ▶); software used to prepare material for publication: *publCIF* (Westrip, 2010 ▶).

## Supplementary Material

Crystal structure: contains datablock(s) I, global. DOI: 10.1107/S1600536812037622/wm2677sup1.cif

Structure factors: contains datablock(s) I. DOI: 10.1107/S1600536812037622/wm2677Isup2.hkl

Additional supplementary materials:  crystallographic information; 3D view; checkCIF report

## Figures and Tables

**Table 1:** Hydrogen-bond geometry (Å, °)

*D*—H⋯*A*	*D*—H	H⋯*A*	*D*⋯*A*	*D*—H⋯*A*
O*W*5—H⋯O1	0.70 (3)	2.18 (2)	2.875 (2)	170 (3)

**Fig. 1.**
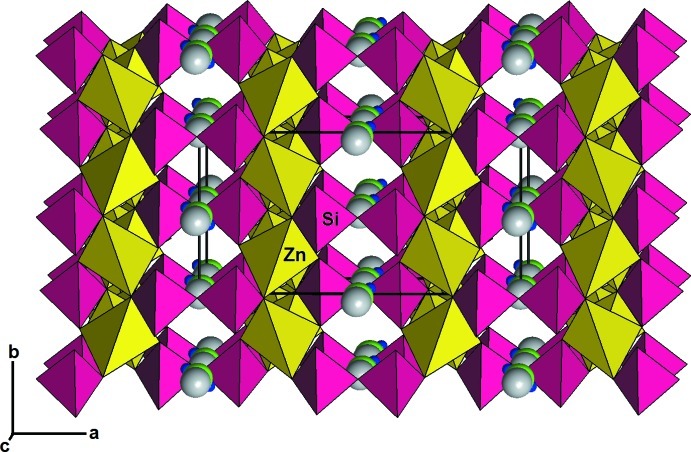
Crystal structure of junitoite. The gray, green, and small blue spheres represent Ca, Ow5, and H atoms, respectively. The yellow and red tetrahedra represent ZnO4 and SiO4 groups, respectively.

**Fig. 2.**
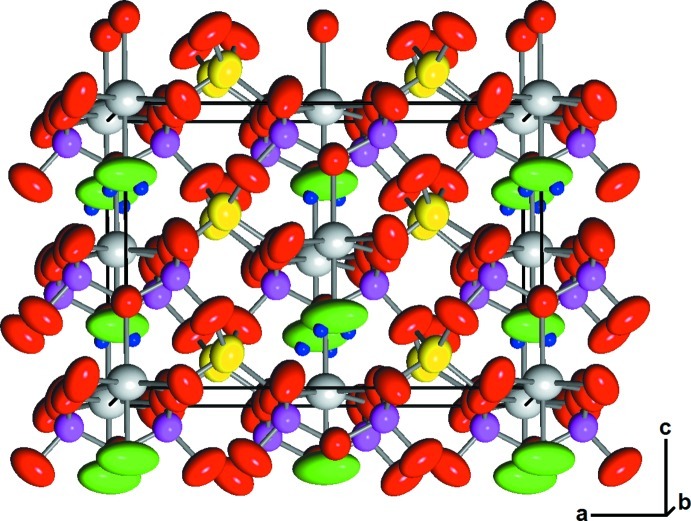
Atoms in junitoite with corresponding ellipsoids at the 99.9% probability level. The gray, yellow, purple, red, and green ellipsoids represent Ca, Zn, Si, O, and Ow5, respectively. The small blue spheres represent H atoms.

## supplementary crystallographic information

### Comment

Junitoite, CaZn_2_Si_2_O_7_.H_2_O, from the Christmas Mine, Gila County, Arizona was first described by Williams (1976) with orthorhombic symmetry in space group *Bbm*2 (non-standard setting of space group No. 40) and unit-cell parameters *a* = 6.309, *b* = 12.503, *c* = 8.549 Å. By adopting the standard unit-cell setting of this space group in *Ama*2 (*a* = 12.510, *b* = 6.318, *c* = 8.561 Å) for this mineral, Hamilton & Finney (1985) noted that while the Weissenberg photographic data pointed to *Ama*2, the X-ray diffractometer data were also compatible with the space group *Aea*2. Although the two space groups yielded similar reliability factors *R*1 ~ 0.10 with isotropic displacement parameters for all atoms (H atoms were not located), Hamilton & Finney (1985) chose *Ama*2 for their final structure report because it "produces less distortion of the coordination polyhedra and provides a structure in which the site symmetry of the cations is more similar to other zinc silicates". Their attempts at refinement with anisotropic displacement parameters resulted in non-positive definite displacement parameters for a number of atoms. In our efforts to understand the hydrogen bonding environments in minerals and their relationships to Raman spectra, we concluded that the structural model for junitoite needed improvement. This study reports a structure redetermination of junitoite from the type locality by means of single-crystal X-ray diffraction data, demonstrating that the space group of this mineral is actually *Aea*2, rather than *Ama*2.

The crystal structure of junitoite consists of chains of corner-sharing ZnO_4_ tetrahedra parallel to the *b* axis, cross-linked by Si_2_O_7_ tetrahedral dimers along *a* and *c* to form a three-dimensional framework. The Ca^2+^ cations, situated in cavities of the framework, are bonded to five O atoms and one H_2_O molecule in a distorted octahedral [CaO_5_(H_2_O)] coordination environment (Figs. 1, 2). As described below, it may be useful to consider that there is a Ca—H_2_O bonded pair in the cavity. The structure of junitoite in space group *Aea*2 resembles that in space group *Ama*2 (Hamilton & Finney, 1985). In fact, as noted by Hamilton & Finney (1985), the structure model in *Aea*2 can be attained simply by shifting the origin of the structure model in *Ama*2 from (*x, y, z*) to (*x* - 1/4, *y* - 1/4, *z*). Upon this shift, the only major structural change is that the two unique Zn atoms at the 4*a* sites in the *Ama*2 structure model are transformed into a single atom at the 8*b* site in the *Aea*2 structure model. The numbers and coordination polyhedra of the distinct Ca, Si, and O sites remain unaffected. However, some bond lengths are noticeably different between the two structure models. For example, the Si—O, Zn—O, and Ca—O bond lengths range from 1.55 (5) to 1.69 (5) Å, 1.93 (4) to 1.99 (4) Å, and 2.29 (7) to 2.44 (5) Å in the *Ama*2 structure model, respectively, but from 1.6130 (14) to 1.6719 (12) Å, 1.9454 (13) to 1.9691 (13) Å, and 2.286 (2) to 2.439 (2) Å in the *Aea*2 structure model. The Si—O—Si angle within the Si_2_O_7_ disiilicate group is 124.8 (1)° in our study, which is slightly greater than that (122.4°) determined by Hamilton & Finney (1985).

The hydrogen bond in junitoite is found between Ow5 and O1, with Ow5 as the donor and O1 as the acceptor. This agrees with the calculated bond-valence sums of 0.42 valence units for Ow5 and 1.77 valence units for O1 by using the parameters given by Brese & O'Keeffe (1991). For numerical details of the hydrogen-bonding geometry, see: Table 1.

Remarkably, junitoite is topologically related to a group of compounds with the general formula Ba*M*^2+^_2_Si_2_O_7_, where *M* = Be (barylite and clinobarylite), Fe (andremeyerite), Cu (scottyite), and Mg, Mn, Co, and Zn in synthetic phases. These Ba-silicates are all comprised of corner-sharing *M*O_4_ tetrahedral chains that are interlinked by Si_2_O_7_ tetrahedral dimers and Ba^2+^ cations, despite their diverse structural symmetries (Yang *et al.*, 2012). Intriguingly, there is no documentation for any Sr*M*_2_Si_2_O_7_ compounds. It then begs the question whether the Ba*M*_2_Si_2_O_7_ compounds are capable of accommodating a significant amount of cations smaller than Ba^2+^. Similar to the pair (Ca^2+^ + H_2_O) in junitoite, the Ba^2+^ cations in the Ba*M*_2_Si_2_O_7_ structures are also situated in the cavities of the framework formed by the Si_2_O_7_ dimers and the *M*O_4_ tetrahedral chains. Conceivably, any substantial replacement of large Ba^2+^ by smaller divalent cations (such as Sr^2+^) would require, in addition to the other structural adjustments (such as the tilting or distortion of *M*O_4_ and/or SiO_4_ tetrahedra), a further narrowing of the Si—O—Si angle in the Si_2_O_7_ group in order to satisfy the bonding environment for smaller cations. This, however, would not be energetically favorable, because the Si—O—Si angles in the Ba*M*_2_Si_2_O_7_ compounds, ranging from 124 to 135°, are already among the smallest of disilicate materials, *e.g.* for high-temperature BaZn_2_Si_2_O_7_ (Lin *et al.*, 1999), high-pressure rare earth (*RE*) disilicates *RE*_2_Si_2_O_7_ (Fleet & Liu, 2001) or BaKY(Si_2_O_7_) (Kolitsch *et al.*, 2009). Accordingly, any sizable substitution of smaller Sr^2+^ for Ba^2+^ would worsen the bonding energetics for this site and thus destabilize the structure. For junitoite, Ca^2+^ by itself, which is even smaller than Sr^2+^, is apparently too small to occupy the cavities in the framework. Therefore, the presence of the H_2_O—Ca^2+^ bonded pair is essential to stabilize its structure. By the same token, one could argue that the pair (Sr^2+^ + H_2_O) together may be too large for the cavities in the structures analogous to those for the Ba*M*_2_Si_2_O_7_ materials, since there is no report for any Sr*M*^2+^_2_Si_2_O_7_.H_2_O compound up to date. Based on this reasoning, we postulate that more compounds with composition Ca*M*^2+^_2_Si_2_O_7_.H_2_O may be found in nature or synthesized in laboratories. Furthermore, it would be interesting if the Sr—H_2_O pair might be found in digermanates, where this structural unit is even larger.

### Experimental

The junitoite crystal used in this study is from the type locality, the Christmas Mine, Gila County, Arizona and is in the collection of the RRUFF project (http://rruff.info/R120100). Its chemical composition measured by Williams (1976) is Ca_0.98_Zn_1.96_Si_1.84_O_6.6_.1.13H_2_O.

### Refinement

The H atom was located near Ow5 from difference Fourier syntheses and its position refined freely with a fixed isotropic displacement (*U_iso_* = 0.03). For simplicity, the ideal chemistry, CaZn_2_Si_2_O_7_.H_2_O, was assumed during the final refinement. The highest residual peak in the difference Fourier maps was located at (0.6270, 0.1150, 0.9598), 0.69 Å from O1, and the deepest hole at (0.3072, 0.2596, 0.0874), 0.81 Å from Zn.

### Crystal data

**Table:**

CaZn_2_Si_2_O_7_·H_2_O	*F*(000) = 696
*M**_r_* = 357.02	*D*_x_ = 3.506 Mg m^−^^3^
Orthorhombic, *A**e**a*2	Mo *K*α radiation, λ = 0.71073 Å
Hall symbol: A 2 -2ac	Cell parameters from 1387 reflections
*a* = 12.530 (4) Å	θ = 4.3–33.2°
*b* = 6.3056 (18) Å	µ = 8.21 mm^−^^1^
*c* = 8.562 (3) Å	*T* = 293 K
*V* = 676.5 (3) Å^3^	Cuboid, colorless
*Z* = 4	0.06 × 0.06 × 0.05 mm

### Data collection

**Table:**

Bruker APEXII CCD area-detector diffractometer	1257 independent reflections
Radiation source: fine-focus sealed tube	1189 reflections with *I* > 2σ(*I*)
Graphite monochromator	*R*_int_ = 0.021
φ and ω scan	θ_max_ = 33.3°, θ_min_ = 3.3°
Absorption correction: multi-scan (*SADABS*; Sheldrick, 2005)	*h* = −8→19
*T*_min_ = 0.639, *T*_max_ = 0.684	*k* = −9→8
2719 measured reflections	*l* = −12→13

### Refinement

**Table:**

Refinement on *F*^2^	Hydrogen site location: difference Fourier map
Least-squares matrix: full	H atoms treated by a mixture of independent and constrained refinement
*R*[*F*^2^ > 2σ(*F*^2^)] = 0.017	*w* = 1/[σ^2^(*F*_o_^2^) + (0.0094*P*)^2^] where *P* = (*F*_o_^2^ + 2*F*_c_^2^)/3
*wR*(*F*^2^) = 0.044	(Δ/σ)_max_ = 0.001
*S* = 1.07	Δρ_max_ = 0.52 e Å^−^^3^
1257 reflections	Δρ_min_ = −0.65 e Å^−^^3^
65 parameters	Extinction correction: *SHELXL97* (Sheldrick, 2008), Fc^*^=kFc[1+0.001xFc^2^λ^3^/sin(2θ)]^-1/4^
1 restraint	Extinction coefficient: 0.0120 (4)
Primary atom site location: structure-invariant direct methods	Absolute structure: Flack (1983), 580 Friedel pairs
Secondary atom site location: difference Fourier map	Flack parameter: 0.023 (12)

### Special details

**Table:**

Geometry. All e.s.d.'s (except the e.s.d. in the dihedral angle between two l.s. planes) are estimated using the full covariance matrix. The cell e.s.d.'s are taken into account individually in the estimation of e.s.d.'s in distances, angles and torsion angles; correlations between e.s.d.'s in cell parameters are only used when they are defined by crystal symmetry. An approximate (isotropic) treatment of cell e.s.d.'s is used for estimating e.s.d.'s involving l.s. planes.
Refinement. Refinement of *F*^2^ against ALL reflections. The weighted *R*-factor *wR* and goodness of fit *S* are based on *F*^2^, conventional *R*-factors *R* are based on *F*, with *F* set to zero for negative *F*^2^. The threshold expression of *F*^2^ > σ(*F*^2^) is used only for calculating *R*-factors(gt) *etc*. and is not relevant to the choice of reflections for refinement. *R*-factors based on *F*^2^ are statistically about twice as large as those based on *F*, and *R*- factors based on ALL data will be even larger.

### Fractional atomic coordinates and isotropic or equivalent isotropic displacement parameters (Å^2^)

**Table:**

	*x*	*y*	*z*	*U*_iso_*/*U*_eq_	
Ca	0.0000	0.5000	0.51882 (6)	0.00899 (12)	
Zn	0.252164 (9)	0.244531 (17)	0.13494 (6)	0.00808 (7)	
Si	0.11827 (4)	0.00274 (4)	0.39406 (7)	0.00615 (9)	
O1	0.12311 (9)	0.2992 (2)	0.01003 (15)	0.0107 (3)	
O2	0.12739 (10)	0.21991 (19)	0.49272 (17)	0.0099 (3)	
O3	0.20596 (13)	−0.00482 (13)	0.25347 (15)	0.0101 (3)	
O4	0.0000	0.0000	0.3037 (2)	0.0082 (3)	
OW5	0.0000	0.5000	0.2518 (3)	0.0209 (7)	
H	0.035 (3)	0.448 (4)	0.200 (3)	0.030*	

### Atomic displacement parameters (Å^2^)

**Table:**

	*U*^11^	*U*^22^	*U*^33^	*U*^12^	*U*^13^	*U*^23^
Ca	0.0106 (2)	0.0078 (2)	0.0085 (2)	0.00019 (13)	0.000	0.000
Zn	0.00755 (10)	0.00739 (10)	0.00931 (11)	−0.00067 (7)	0.00070 (15)	−0.00072 (18)
Si	0.00563 (18)	0.00687 (18)	0.00596 (19)	−0.00022 (11)	0.00019 (16)	0.00035 (16)
O1	0.0084 (5)	0.0112 (5)	0.0126 (8)	−0.0010 (4)	−0.0020 (4)	0.0046 (5)
O2	0.0086 (5)	0.0109 (5)	0.0102 (8)	0.0019 (4)	−0.0026 (4)	−0.0035 (5)
O3	0.0124 (7)	0.0080 (6)	0.0099 (6)	−0.0001 (3)	0.0045 (4)	0.0003 (4)
O4	0.0063 (8)	0.0112 (8)	0.0071 (9)	0.0000 (4)	0.000	0.000
OW5	0.0272 (15)	0.0278 (18)	0.0076 (11)	0.0100 (7)	0.000	0.000

### Geometric parameters (Å, º)

**Table:**

Ca—OW5	2.286 (2)	Zn—O3^v^	1.9501 (11)
Ca—O2^i^	2.3910 (12)	Zn—O3	1.9589 (11)
Ca—O2	2.3910 (12)	Zn—O1	1.9691 (13)
Ca—O1^ii^	2.4381 (13)	Si—O2	1.6130 (14)
Ca—O1^iii^	2.4381 (13)	Si—O1^vi^	1.6239 (14)
Ca—O4^ii^	2.439 (2)	Si—O3	1.6305 (16)
Zn—O2^iv^	1.9454 (13)	Si—O4	1.6719 (12)
			
OW5—Ca—O2^i^	84.64 (4)	O1^iii^—Ca—O4^ii^	91.77 (4)
OW5—Ca—O2	84.64 (4)	O2^iv^—Zn—O3^v^	100.47 (6)
O2^i^—Ca—O2	169.28 (8)	O2^iv^—Zn—O3	119.31 (6)
OW5—Ca—O1^ii^	88.23 (4)	O3^v^—Zn—O3	117.42 (7)
O2^i^—Ca—O1^ii^	81.25 (6)	O2^iv^—Zn—O1	108.13 (8)
O2—Ca—O1^ii^	98.41 (6)	O3^v^—Zn—O1	111.21 (6)
OW5—Ca—O1^iii^	88.23 (4)	O3—Zn—O1	100.32 (6)
O2^i^—Ca—O1^iii^	98.41 (6)	O2—Si—O1^vi^	110.37 (11)
O2—Ca—O1^iii^	81.25 (6)	O2—Si—O3	111.33 (7)
O1^ii^—Ca—O1^iii^	176.46 (7)	O1^vi^—Si—O3	113.77 (7)
OW5—Ca—O4^ii^	180.0	O2—Si—O4	108.30 (6)
O2^i^—Ca—O4^ii^	95.36 (4)	O1^vi^—Si—O4	107.94 (6)
O2—Ca—O4^ii^	95.36 (4)	O3—Si—O4	104.79 (10)
O1^ii^—Ca—O4^ii^	91.77 (4)		

Symmetry codes: (i) −*x*, −*y*+1, *z*; (ii) *x*, *y*+1/2, *z*+1/2; (iii) −*x*, −*y*+1/2, *z*+1/2; (iv) −*x*+1/2, *y*, *z*−1/2; (v) −*x*+1/2, *y*+1/2, *z*; (vi) *x*, *y*−1/2, *z*+1/2.

### Hydrogen-bond geometry (Å, º)

**Table:**

*D*—H···*A*	*D*—H	H···*A*	*D*···*A*	*D*—H···*A*
O*W*5—H···O1	0.70 (3)	2.18 (2)	2.875 (2)	170 (3)
